# A female presenting with prolonged fever, weakness, and pain in the bilateral pelvic region: a case report

**DOI:** 10.1186/1757-1626-2-194

**Published:** 2009-11-16

**Authors:** Tufan Tasci, Beyazit Zencirci

**Affiliations:** 1 Department of Surgery, Mostas Private Health Hospital, Kahramanmaras, Turkey; 2 Department of Anesthesiology and Reanimation, Mostas Private Health Hospital, Kahramanmaras, Turkey

## Abstract

**Introduction:**

Psoas abscess, a collection of pus in the iliopsoas compartment that has traditionally been classified as primary and secondary according to its origin.

**Case presentation:**

48-year-old Turkish female presented to the department with fever, weakness and pain in the bilateral pelvic region. In contrast abdominal magnetic resonance, a collection compatible with the hyperintense abscess was observed in the right and left ilipsoas muscles. It was decided to simultaneously drain both abscesses of the case who had been using oral and intravenous broad-spectrum antibiotics for two months. No factors were detected in the microbiological reviews made on the abscess fluid of the operated case. The case was also examined in terms of tuberculosis and Crohn's disease and no findings were encountered to rise suspicions of such diseases.

**Conclusion:**

An abscess of the psoas muscle was a rare entity. However, with the increased use of computed tomography scans to evaluate patients with unknown foci of sepsis, psoas abscesses now are diagnosed and reported more frequently. What should be done after diagnosis are, if possible, defining the infection factor, selecting the appropriate antibiotics and draining the abscess openly or percutaneously.

## Introduction

The psoas muscle is a retroperitoneal muscle that originates from the lateral borders of the 12th thoracic to 5th lumbar vertebrae and inserts in the lesser trochanter of the femur. The psoas muscle lies in close proximity to many other organs, including the sigmoid colon, jejunum, appendix, ureters, aorta, renal pelvis, pancreas, iliac lymph nodes, and spine. Thus, infections in these organs can contiguously spread to the psoas muscle. The psoas muscle has a rich vascular supply that is believed to predispose it to hematogenous spread from sites of occult infection [1].

Symptoms are often nonspecific. Patients may have fever, flank pain, abdominal pain, or limp. Because of the innervation of the psoas muscle by L2, L3, and L4, pain due to inflammation sometimes radiates anterior to the hip and thigh. Other symptoms are nausea, malaise, and weight loss. A good physical examination is critical for the prompt diagnosis of psoas abscess. Laboratory tests are helpful in the evaluation of suspected psoas abscess. Leukocytosis, elevated erythrocyte sedimentation rate (ESR), and elevated blood urea nitrogen (BUN) were reported in 100% of patients in the series from Johns Hopkins [2]. Whenever psoas abscess is suspected, computed tomography (CT) should be done for definitive diagnosis. So CT is diagnostic in 80 to 100% cases of psoas abscess [3].

Treatment involves the use of appropriate antibiotics, as well as drainage of the abscess. It has been suggested that in cases of psoas abscess believed to be primary, antistaphylococcal antibiotic therapy should be started before final bacteriologic diagnosis. But the identification of non-staphylococcus organisms in some patients to start treatment with broad spectrum antibiotics pending final bacteriologic diagnosis [2]. Drainage of the abscess may be done as a surgical drainage or through CT-guided percutaneous drainage. Antibiotics are sometimes continued up to two weeks after complete drainage of the abscess.

## Case presentation

A 48-year-old Turkish female case was suffering from fever, weakness, and pain in the bilateral pelvic region fort the last 3 months. Therefore, the patient was applied different medical treatments including use of broad-spectrum antibiotics (ceftriaxone, ciprofloxacin, gentamicin, rifampicin, etc), but the complaints were not resolved.

Abdominal examination revealed only moderate tenderness in the bilateral flanks but no masses and the abdomen was soft and relax. But she had bilateral physical signs of psoas inflammation (Maneuvers to test for iliopsoas inflammation - Place your hand just proximal to the patient's ipsilateral knee. Then ask the patient to raise his thigh against your hand. This causes contraction of the psoas against resistance and leads to pain. With patient lying on the unaffected side, hyperextension of the affected hip results in pain due to stretching of the psoas muscle).

**Figure 1:**
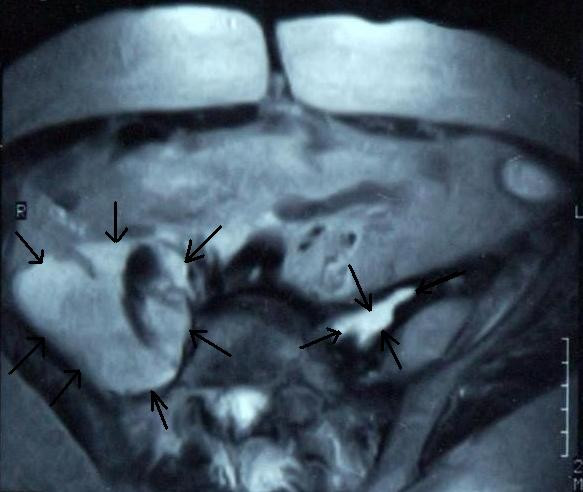
**Abdominal MRI showing bilateral psoas muscle abscesses (arrows)**.

Laboratory blood test results were in the normal range except for a white blood cell count of 16,3 × 10^9^/L and ESR 60 mm/hour. Plain X-ray was normal. However, in the contrast abdomen MRI, a collection compatible with the hyperintense abscess was detected in the right ilipsoas muscle as 9 × 7 cm and in the left psoas muscle as 5 × 2 cm (Figure 1).

The case was considered as a primary psoas abscess and was hospitalized in the general surgery service. Comprehensive biochemical tests (including tumor markers, collagen tissue diseases, tuberculosis etc.) were carried out on the patient. Blood cultures (for fungi, aerobic, anaerobic bacteria and mycobacteria) and urine cultures were taken. In the meanwhile, although endoscopic and colonoscopic examinations were carried out (in order to detect unspotted diseases such as tumoral formations, Crohn's etc.), no results were obtained. It was decided to perform a surgical open drainage on the abscess upon detecting that there were no laboratory findings to prove the presence of a problem with immune system and seeing that there was no reproduction according to blood culture results (culture results were negative).

Surgery was done via McBurney incision towards lumbar region, muscle splitting, extra-peritoneally, and the peritoneum was pushed medially until exposure of the right psoas muscle. The muscle was first aspirated then incised and 320 ml of purulent material which impressioned *Staphylococcus aureus *was drained. A large hemovac drain was inserted into the incised portion of the right psoas muscle and fixed to the skin via a separate incision. The same surgical procedure was performed in the left psoas muscle by positioning the patient. The left psoas muscle was first aspirated then incised and 110 ml of purulent material which impressioned *Staphylococcus aureus *was drained too (dark white coloured, partly sticky and malodorous). During the operation, separate cultures (for fungi, aerobic, anaerobic bacteria and mycobacteria) were taken from both psoas abscesses (both from the abscess wall and pouch). The wound was closed in layers. Post-operatively, antistaphylococcal antibiotic therapy was started to continue for two weeks. Postoperatively, the patient improved dramatically and the post-operative period was uneventful.

**Figure 2:**
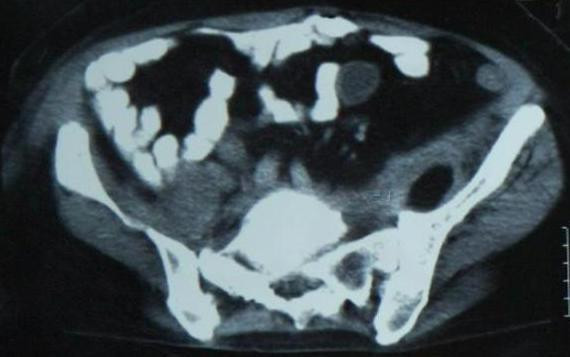
**In the abdominal CT, no abscess observed in the right and left psoas muscles**.

Follow-up CT showed substantial reduction in the size of the collection. The drain was removed after 7 days. The wound was dry and clean. In the last control CT, it was seen that the abscess formation was completely removed [Figure 2]. Therewith, the case was discharged from the hospital on the ninth post-operative day.

## Discussion

The iliopsoas compartment is an extraperitoneal space which contains the iliopsoas and iliacus muscles. The psoas muscle lies in close proximity to organs such as the sigmoid colon, appendix, jejunum, ureters, abdominal aorta, kidneys, pancreas, spine, and iliac lymph nodes. Hence infections in these organs can spread to the iliopsoas muscle [4]. On the other hand abundant blood supply of the muscle is believed to predispose it to haematogenous spread from occult sites of infection [2].

A primary psoas abscess (PA) occurs from haematogenous dissemination of a distant infection and is now the predominant form [1,2]. A secondary abscess arises by contiguous spread of a local infective process and inflammatory or neoplastic diseases of the bowel, kidney and spine, such as Crohn's disease and appendicitis, contribute the majority of secondary cases [2]. Impairment of immunocompetence by infection, iatrogenic immunosuppression or following surgery predisposes to IPA formation - particularly primary cases.

The clinical presentation of PA is often variable and can be non-specific. The classical clinical triad consisted fever, back pain and limp. Other symptoms are vague abdominal pain, malaise, nausea and weight loss [4]. But classical clinical symptoms are rarely present in its entirety and prompt diagnosis continues to rely upon retaining a high degree of suspicion as the signs and symptoms may be diffuse chronic and nonspecific.

Plain film radiography, ultrasonography, CT scan, magnetic resonance imaging (MRI) or FDG-positron emission tomography (PET) may be used to diagnose these infections [5,6]. However, CT scan is considered the 'gold standard' for definitive diagnosis [2,4]. Some authors believe that MR images are superior to CT scans because of better discrimination of soft tissues and the ability to visualize the abscess wall and the surrounding structures without using an i.v. contrast medium. But Gallium-67 scanning is also an effective method of detecting inflammatory lesions, especially abscesses [6].

Other laboratory findings include anemia, an elevation of the white blood cell count, and increases in the C-reactive protein (CRP) level and erythrocyte sedimentation rate (ESR). There was an increase in the leukocytosis and erythrocyte sedimentation rates of our case. Blood cultures may be positive and pus culture via image-guided or surgical aspiration should be carried out. No bacteria were found in samples (either blood or pus) from our patient. We believe that was a result of the case's previous use of various broad-spectrum antibiotics for treatment purposes.

The microbiology of PAs depends on a primary or secondary aetiology. In 1° PA *Staphylococcus aureus *is the predominant organism, although infections from Pseudomonas, Haemophilus, and Proteus species are also reported [2]. The bacteriology of secondary PA usually reflects the underlying condition and enteric organisms (Escherichia coli, Enterobacter and Salmonella) predominate [7]. The purulent abscess that appeared as Staphylococcus aureus was drained from both psoas muscles of our case.

Unfortunately, whilst the isolation of enteric organism strongly suggests a secondary abscess, cases of primary abscess yielding gut organisms and conversely *Staph. aureus *from secondary abscesses have both been reported [8,9]. In areas where tuberculosis remains a concern, mycobacterial infection must be considered.

The treatment of a psoas abscess involves the use of appropriate antibiotics along with drainage of the abscess. Patients with a suspected primary psoas abscess should be treated with antibiotics as an empirical treatment even before the culture results are known. The antibiotics we used can be considered an empirical treatment as the culture results were negative. In secondary psoas abscesses, broad spectrum antibiotics (*covering both aerobic and anaerobic bacteria*) should be considered [2].

Drainage of the abscess should be performed. It may be carried out through surgical drainage or image-guided percutaneous drainage (PCD) but hospital stay is significantly longer compared with open drainage [2]. On the other hand, literature, however, has been inconsistent as to whether open or percutaneous drainage is more effective [10].

Secondary abscesses are likely to need definitive surgery to treat the underlying cause and if borne in mind when planning the initial treatment, open drainage can be combined with definitive surgery, minimizing hospital stay and the need for further admissions.

Psoas abscess is a rare situation with a nonstable and non-unique clinic which makes it easier to misdiagnose or to make late diagnoses. In the event of infection with an undetected source or prolonged fever, as well as abdominal solid organs, peritoneal cavity and retroperitoneal region should also be carefully examined. It should be noted that unnecessary use of antibiotics that is started without the required examinations will not only delay diagnosis and treatment but also effect the culture results. Abscess drainage along with antibiotic treatment constitute the basis of this treatment.

## Competing interests

The authors declare that they have no competing interests.

## Authors' contributions

BZ presented the case history, performed case management, drafted the manuscript; TT carried out the operation of the case and participated in the patient's management. All authors read and approved the final manuscript.

## Consent

Written informed consent was obtained from the patient's for the publication of this case report and accompanying images. A copy of the written consent is available for review by the Editor-in-Chief of this journal.
